# 骨肉瘤肺转移存在诊断治疗不足——中国骨肉瘤肺转移多学科诊疗现况调查

**DOI:** 10.3779/j.issn.1009-3419.2016.03.06

**Published:** 2016-03-20

**Authors:** 晓征 康, 真 黄, 安辉 石, 洁 王, 冬梅 林, 应实 孙, 广迎 朱, 晓辉 牛, 克能 陈

**Affiliations:** 1 100142 北京，北京大学肿瘤医院暨北京市肿瘤防治研究所胸外一科 Key Laboratory of Carcinogenesis and Translational Research (Ministry of Education), the First Department of Thoracic Surgery, Peking University Cancer Hospital and Institute, Peking University School of Oncology, Beijing 100142, China; 2 恶性肿瘤发病机制及转化研究教育部重点实验室；100035 北京，北京积水潭医院骨肿瘤科 Department of Orthopedic Oncology, Beijing Jishuitan Hospital, Peking University, Beijing 100035, China; 3 100142 北京，北京大学肿瘤医院暨北京市肿瘤防治研究所放疗科 Key Laboratory of Carcinogenesis and Translational Research (Ministry of Education), Department of Radiation Oncology, Peking University School of Oncology, Beijing 100142; 4 100142 北京，北京大学肿瘤医院暨北京市肿瘤防治研究所胸内一科 the First Department of Thoracic Medical Oncology, Peking University School of Oncology, Beijing 100142; 5 100142 北京，北京大学肿瘤医院暨北京市肿瘤防治研究所病理科 Department of Pathology, Peking University School of Oncology, Beijing 100142; 6 100142 北京，北京大学肿瘤医院暨北京市肿瘤防治研究所医学影像科 Department of Radiology, Peking University Cancer Hospital and Institute, Peking University School of Oncology, Beijing 100142, China

**Keywords:** 骨肉瘤, 肺转移, 实践模式, Osteosarcoma, Pulmonary metastases, Practice pattern

## Abstract

**背景与目的:**

骨肉瘤是最常见的骨原发恶性肿瘤，好发于青少年。尽管多学科框架下骨肉瘤的治疗已经取得瞩目成就，然而影响骨肉瘤远期生存的重要因素是肺转移。约有1/2以上患者在其病程的不同时期会发生肺转移，其中10%~15%为同时性肺转移。毋庸置疑，治疗肺转移瘤的首选方法为外科切除，但因骨肉瘤肺转移的诊疗涉及多学科多专业，而由于各学科之间的理念存在差异，因此尚未形成标准化的诊疗模式。本研究通过问卷调查方式旨在了解当前我国骨肉瘤大型医疗中心在此领域中的诊疗模式现况。

**方法:**

2015年9月-2015年11月期间，一项关于骨肉瘤肺转移的诊疗现况问卷在全国骨肉瘤大型诊疗中心范围内开展。根据调查对象所在单位、工作年限、年均收治病例数及医学专业不同，进一步行分层分析。

**结果:**

调查共发放问卷150份，回收有效问卷105份，总应答率为70%。首先，关于骨肉瘤肺转移治疗的核心问题，41.0%的调查对象认同骨肿瘤外科专业在骨肉瘤肺转移多学科诊疗实践中的主导地位；首选肺转移瘤切除术作为转移灶局部控制措施的比例约64%；78.1%的调查对象会推荐患者行肺转移瘤切除术；骨科及胸外科医师较之其他专业更加积极（同骨科专业相比，OR_其他专业_=0.02；95%CI：0.00-0.22；*P*=0.001）；胸外科医师更倾向根据经验决定肺转移瘤切除术的指证，而非受限于病灶具体数目（OR_胸外科专业_=20.93；95%CI：2.05-213.64；*P*=0.001）。其次，关于骨肉瘤肺转移影像学诊断方法，约83%首选胸部计算机断层扫描（computed tomography, CT）；约70%的调查对象推荐原发灶切除术后3个月行胸部CT随诊；约68%认为CT诊断准确度90%水平；约92%同时推荐行肺外影像学评估。最后，关于术后生存获益的定义问题，约46%认为肺转移瘤术后生存期超过6个月即为生存获益。

**结论:**

本次问卷调查提供了我国骨肉瘤肺转移诊疗模式的现况信息，其反映出缺少统一规范诊疗模式的不足。调查结果为今后研究方向及国际性临床指南提供了基线数据。

## Abstract (en)

**Background and objective:**

Osteosarcoma is the most frequent primary cancer of bone. The incidence is higher in adolescents. Large improvement, though, has been made in the treatment of osteosarcoma under the framework of multidisciplinary team, an important prognostic factor for osteosarcoma is pulmonary metastasis. Surgical resection of lung metastases is widely accepted as the optimal modality in osteosarcoma patients. Undoubtedly, surgical resection of lung metastases is widely accepted as the optimal modality in osteosarcoma patients. However, since current conceptions within the surgical approach to lung metastasectomy involve multidisciplinary collaboration, which are highly variable, there is not neither consensus nor standardized practice patterns. We conduct a survey aiming to reveal areas of consistency in current clinical practice on lung metastasectomy among Chinese osteosarcoma high volume centers.

**Methods:**

A questionnaire survey specific to the nationwide high volume centers of osteosarcoma was conducted from September 2015 to November 2015. Analyses were stratified by hospital, working duration, case volume and medical discipline.

**Results:**

Of 150 invited physicians, 105 participated, resulting in an overall response rate of 70%. Forty-one percent of the responded physicians agreed with the statement that orthopedic oncology should play a predominant role in the multidisciplinary team. More than 64% of respondents chose metastasectomy as the preferred local control approach, and up to 78.1% of respondents recommended pulmonary metastasectomy for patients. Compared with orthopedic surgeons, other physicians were significantly more likely to report not advocating pulmonary metastasectomy in their practice (OR_others_=0.02; 95%CI: 0.00-0.22; *P*=0.001), and thoracic surgeons were more likely to decide metastasectomy according to indications rather than the number limit of metastases (OR_thoracic surgeons_=20.93; 95%CI: 2.05-213.64; *P*=0.001). For the most preferred radiographic evaluation option, approximately 83% of respondents reported diagnostic use of computed tomography (CT). More than 70% of respondents reported that chest CT follow-up should be 3 months after the primary site resection; approximately 68% deemed the diagnostic accuracy of CT about 90%; most respondents (92%) recommended the extrapulmonary imaging evaluation simultaneously. Around 46% of respondents reported that survival duration longer than 6 months after pulmonary metastasectomy is beneficial.

**Conclusion:**

This study offers new information about the variability in the reported management of pulmonary metastatic osteosarcoma in China, reflecting the deficiencies in unified practice patterns. The results of this survey also provide baseline data for future research and for the development of international guidelines.

骨肉瘤是青少年最常见的骨来源的恶性肿瘤，治疗的好坏不仅影响社会劳动力及社会资源，而且导致许多家庭陷入痛苦。虽然近年骨肉瘤原发瘤的诊断治疗取得了较大进展，但肺转移始终是影响远期疗效难以改善的障碍。约有1/2以上患者在其病程的不同时期会发生肺转移，其中10%-15%为同时性肺转移^[1, 2]^。

骨肉瘤肺转移的诊断治疗涉及骨科、胸外科、肿瘤内科、放射治疗科、影像学及病理学等多专业学科组成，但由于如下原因骨肉瘤肺转移的多学科协作实践并不理想：①骨肉瘤虽然是骨科最常见的恶性肿瘤，但就发病率而言仍属罕见肿瘤，鲜有除骨科以外的专家重视，因此日常各学科接触的病例均为散发病例；②骨肉瘤肺转移诊疗的主要参与学科为胸外科及骨科，但该两专业无论从医师训练及日常联系均相距甚远且无交叉，使得学科之间相互并不了解对方专业的进展；③多学科缺少对肿瘤转移，尤其寡转移知识更新普及，始终固守着一旦发生肺转移即拒绝局部治疗的陈旧观念。北京大学肿瘤医院胸外一科是中国同行业中的顶级团队，团队掌握着最先进的胸外科技术，并有多年形成胸部肿瘤多学科协作机制。而北京大学积水潭医院骨肿瘤科则是全球最大的骨肿瘤专科之一，集中有世界上数量最多的骨肿瘤病例。从2005年以这两专业为主组成骨与软组织肉瘤肺转移多学科协作团队，迄今已治疗超过250例骨肿瘤肺转移患者。据我们所知是这一时段中国国内甚至全球积累病例最多的团队。经仔细阅读文献，我们认为中国骨肉瘤肺转移的诊疗存在不足。为此，我们开展了一项针对我国骨肉瘤肺转移诊治大型医疗中心的问卷调查，旨在了解不同专业之间诊疗策略的现况。

## 1 材料和方法

### 1.1 问卷内容与中心定义

本研究针对当今骨肉瘤肺转移诊疗策略问题制订了一份调查问卷，内容包涵骨肉瘤肺转移诊疗相关问题11项：包括主导治疗的专业学科，影像学评估的首选方法、随诊时间、准确度，是否推荐肺外影像学评估，最佳治疗模式，最佳局部控制疗法与最佳治疗时机，肺转移瘤外科治疗适应证以及判断治疗后生存获益的标准，详见具体见附录。

通过在全国范围向各地大型骨肉瘤诊疗中心的医师发送问卷方式，其中大型骨肉瘤诊疗中心的定义因专业不同而异：骨科年均病例数超过50例；胸外科年均例数超过30例。问卷调查方式采用半结构式，即多数问题答案预先已经确定，由调查对象自行逐一进行勾选；部分选项不设定固定答案，由调查对象自由填写。调查时间范围从2015年9月至11月，计划发出问卷共150份。

### 1.2 统计学方法

问卷结果首先匿名化然后通过Epidata专业软件v2.0.3.15（EpiData Association, Denmark）录入数据库。所有研究项目均以分类变量形式表示，采用*Logistic*回归分析确定医师相关因素与诊疗模式之间的统计学关联。解释变量以比值比（odds ratios, ORs），可信区间（confidence intervals, CIs）及P值表示，*P*＜0.05为差异有统计学意义。所有分析计算选用SAS version 9.4（SAS Institute Inc., Cary, NC, USA）完成。绘图采用GraphPad Prism version 6.01（GraphPad Software Inc., CA, USA）完成。

## 2 结果

### 2.1 一般资料

本研究共发放调查问卷150份，回收有效问卷105份，有效应答率为70%。调查对象专业包括骨科（61例，58.1%），胸外科（26例，24.7%），放疗科（8例，7.6%），肿瘤内科（2例，1.9%），病理科（3例，2.9%）及其他（5例，4.8%）。调查对象从业单位以综合性医院居多（74.3%），而肿瘤专科医院其次（22.9%）。调查对象的中位工作年限为10年-20年，并且中位年均接诊骨肉瘤病例数为11例-30例。指导临床诊疗决策的最常用（39.1%）依据为国际临床指南。调查对象的一般信息见表 1。

**1:** 调查对象一般资料 Demographics

Items	*n*	Proportion (%)
Hospital of current practice		
Academic medical center	78	74.3
Cancer center	24	22.9
Others	3	2.9
Medical discipline		
Orthopedic surgeon	61	58.1
Thoracic surgeon	26	24.8
Radiation oncologist	8	7.6
Medical oncologist	2	1.9
Pathologist	3	2.9
Others	5	4.8
Years from training		
0-5	18	17.1
5-10	24	22.9
10-20	26	24.8
> 20	37	35.2
Number of osteosarcoma patients seen per year		
0-10	50	47.6
11-30	28	26.7
31-50	11	10.5
> 50	16	15.2
Factor that predominantly guides		
Personal clinical practice and experience	23	21.9
Institutional policies/standards	24	22.9
Systematic review and meta-analysis	6	5.7
International guidelines	41	39.1
Others	11	10.5

### 2.2 治疗模式

关于骨肉瘤肺转移诊疗中主导决策的专业，骨肿瘤科居首位（41%），以后依次为胸外科（26%），肿瘤内科（20%），不确定（8%）及放疗科（5%），并且在骨科专业亚组中共识的比例更高（图 1A）。调查对象（64%）选择肺转移瘤切除术作为最佳局部控制方法，并且一旦发现骨肉瘤肺转移推荐患者行肺转移瘤切除术。骨科及胸外科较之其他专业更加积极，选择比例分别超过90%及60%（图 1B）。多因素*Logistic*回归分析结果表明，控制其他混淆因素情况下，调查对象从事的医学专业与是否积极推荐肺转移瘤切除术存在关联（表 2）。同骨科专业相比，除胸外科以外的其他专业调查对象更倾向于保守治疗（OR_其他专业_=0.02，95%CI: 0.00-0.22，*P*=0.001）。关于肺转移瘤切除术的时机与适应证，不同专业之间的选择变异度较明显（图 1C）。超过52%的调查对象认为适宜手术的病例肺转移瘤病灶数目不应超过5枚，而胸外科专业医师则更多选择由本专业医师决定手术切除病灶数上限（OR_胸外科_=20.93，95%CI: 2.05-213.64，*P*=0.001），详见表 2。最佳治疗模式选择的变异度同样较大（图 1D）。

**1:**
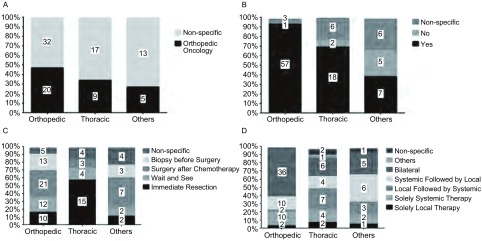
不同专业关于治疗相关问题的调查结果。A：多学科协作中的主导专业；B：推荐支持肺转移瘤切除术；C：肺转移瘤切除术的最佳时机；D：最佳治疗模式。直方图中各个数字代表人数。 Variability in the reported (A) the leading role in multidisciplinary team, (B) the advocacy of pulmonary metastasectomy, (C) the optimal timing pulmonary metastasectomy, and (D) the best therapeutic modality according to medical discipline. Values within bars represent number of responders.

**2:** 临床实践模式相关因素 Factors associated with clinical management patterns

Items	Advocating pulmonary metastasectomy			Surgical indication decided by thoracic surgeon	
	OR (95%CI)	*P*		OR (95%CI)	*P*
Location of current practice					
Academic medical center	1 [Reference]			1 [Reference]	
Cancer center	0.43 (0.09-1.99)	0.282		0.43 (0.08-2.15)	0.301
Others	ND	0.977		6.09 (0.21-175.78)	0.292
Medical discipline						
Orthopedic surgeon	1 [Reference]			1 [Reference]	
Thoracic surgeon	0.22 (0.03-1.94)	0.174		20.93 (2.05-213.64)	0.010
Others	0.02 (0.00-0.22)	0.001		1.18 (0.17-8.04)	0.863
Years from training						
0-5	1 [Reference]			1 [Reference]	
5-10	1.53 (0.17-14.13)	0.709		ND	0.957
10-20	6.93 (0.51-93.81)	0.145		ND	0.946
> 20	2.85 (0.30-26.79)	0.361		ND	0.950
Number of osteosarcoma patients seen per year					
0-10	1 [Reference]			1 [Reference]	
11-30	1.17 (0.18-7.47)	0.871		1.21 (0.14-10.31)	0.861
31-50	0.49 (0.04-6.75)	0.591		3.00 (0.23-39.31)	0.402
> 50	5.02 (0.26-98.47)	0.288		1.58 (0.12-20.28)	0.724
Factor that predominantly navigates clinical management decision					
International guidelines (*e.g*., NCCN)	1 [Reference]			1 [Reference]	
Personal clinical practice and experience	1.70 (0.27-10.64)	0.573		2.22 (0.35-13.98)	0.398
Institutional policies/standards	0.41 (0.08-2.15)	0.294		2.06 (0.47-9.15)	0.341
Systematic literature review and meta-analysis	0.37 (0.03-4.48)	0.437		ND	0.967
Others	0.42 (0.04-4.78)	0.488		1.09 (0.16-7.62)	0.933
ND, not determined; NCCN: National Comprehensive Cancer Network.					

### 2.3 诊断方法

本调查问卷内容涉及关于骨肉瘤肺转移瘤诊断方法选择，约83%的调查对象首选胸部计算机断层扫描（computed tomography, CT）作为骨肉瘤肺转移的评估方法，但是选择何种层厚扫描则在不同专业之间变异度较大（图 2）。骨科医生较之其他专业医生选择5 mm层厚CT扫描的比例较高。总体上超过70%受访者认为骨肉瘤原发灶切除术后3个月即应行胸部CT评估；多数医师（约68%）评价CT诊断准确性超过90%；约92%选择发现肺转移同时需要评估肺外转移灶。

**2:**
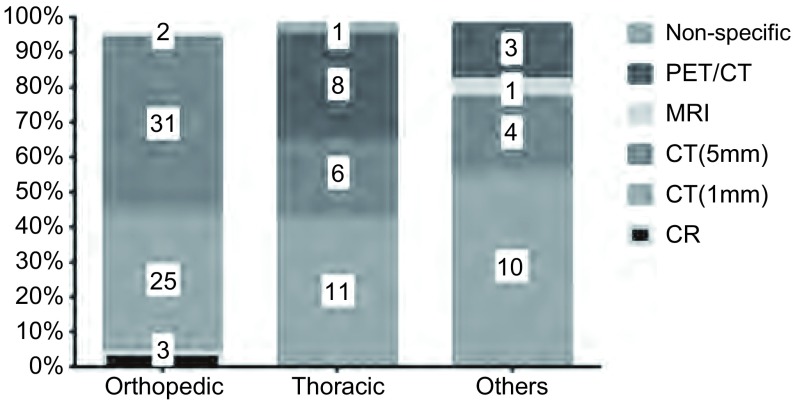
不同专业对于最佳影像学评估方法的调查结果。直方图中各个数字代表人数。 Favored imaging evaluation according to medical discipline. Values within bars represent number of responders. CT: computed tomography; PET: positron emission tomography; CR: computed radiography; MRI: magnetic resource imaging.

### 2.4 治疗价值

关于如何评价骨肉瘤肺转移的治疗价值，约46%的调查对象认为疗后生存期超过6个月即为获益。图 3显示按照不同医院类型，医学专业，工作年限及年均诊治例数进行分层分析的结果。

**3:**
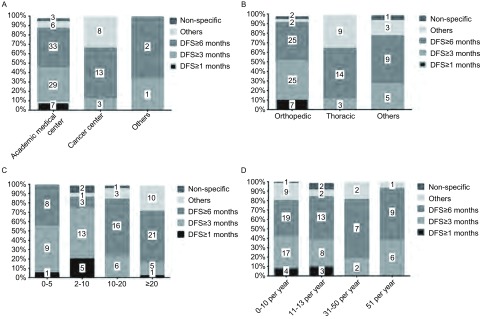
根据不同医院类型（A）、医学专业（B）、工作年限（C）及年均病例数（D），关于术后生存获益定义的调查结果。直方图中各个数字代表人数。 Variability in the reported survival benefits according to (A) hospital, (B) medical discipline, (C) working duration and (D) volume. Values within bars represent number of responders. DFS: disease free survival.

## 3 讨论

关于肺转移瘤外科治疗策略的问卷调查早在2008年即在欧洲胸外科学会开展^[3]^，随后成立了专项研究进一步探索^[4]^。然而该调查对象均为胸外科专业，涉及病种繁多，并且临床经验并非来源于亚裔人群。据我们所知，此次调查在中国骨肉瘤肺转移相关专业中尚属首次。本研究结果反映了当前不同专业医学工作者对于骨肉瘤肺转移诊疗策略的差异性，同时也引起大家研究这种少见疾病肺转移瘤的兴趣。

本调查结果表明中国骨肉瘤诊疗需要多学科协作的共识亟待推出。根据欧洲肿瘤内科学会骨肉瘤临床指南，所有相关诊断治疗问题均应由多学科专家团队完成^[5]^。有关肿瘤转移最著名的“种子与土壤”理论出现1889年由Paget提出^[6]^。骨肉瘤肺转移是这一理论的典型例子，即约90%骨肉瘤转移发生在肺。转移理论发展至今已出现了“广泛转移”与“寡转移”的概念，而骨肉瘤肺转移又是“寡转移”的典型例子。理解这两个理论有助于多学科医生对肺转移瘤外科治疗总则的认识，即只要技术可能且治疗后仍有足够肺功能维持生命，就要设法达到完全消融肿瘤，根治治疗包括手术与放疗。将原发性非小细胞肺癌“寡转移”诊疗策略^[7]^移植到骨肉瘤肺转移诊疗领域中指导实践，这是可行且必需的。显然调查结果表明多学科尤其是除骨科与胸外科以外其他专业医生的治疗态度不够积极，存在治疗不足的可能。

骨肉瘤肺转移的术前评估依靠诊断影像学。本调查结果表明多数依赖胸部CT（5 mm或1 mm层厚），并且始终是标准诊断模式。由于既往研究结果表明传统CT（5 mm层厚）扫描对于肺转移瘤漏诊率达25%^[8]^，因此肺转移瘤术前影像学评估应逐渐过渡到薄层CT重建（1 mm-2 mm），这样才能避免开胸术中肺触诊操作。尽管全身正电子发射型计算机断层显像（positron emission computed tomography, PET）/CT（仅10.5%选择术前需要应用）在术前评估中有助于发现肺外转移灶，但是其临床应用的价值仍存在争议，主要原因在于肺内小结节（直径小于1 cm）的诊断敏感度较差，以及医疗费用较高。由于骨肉瘤肺转移几乎只出现在肺部，因而PET/CT诊断价值有限。

我们调查发现超过70%的调查对象选择原发灶切除术后3个月开始行影像学随访。关于影像学随诊时间间隔尚无明确规定，仅临床指南推荐最初2年每2-3个月复查一次；第3-4年每2-4个月复查一次；第5-10年每6个月复查一次^[5]^。

本地调查结果中变异性最大的两项问题为最佳治疗模式及如何定义生存获益，而这些有待于进一步完善临床指南加以规范。迄今最大宗多中心数据经验来自于国际肺转移瘤登记组织（International Registry of Lung Metastases）^[9]^，其四项预后影响因素：根治性切除可能，无病间期时间，肺转移瘤数目及原发灶病理类型，已被广泛接受并视为经典手术适应证。骨科医师对于肺转移瘤切除术的态度最为积极，其次是胸外科，而其他专业医师则相对保守。对于单侧肺转移患者首先推荐直接根治性手术切除，并且既往经验表明即便多次手术切除，超过1/3患者术后可生存5年以上，因此只要肺转移瘤是可切除的则仍推荐多次重复切除^[10]^。二线化疗对骨肉瘤肺转移的临床价值有限，并且尚无公认的化疗方案^[5]^。放射治疗对于肺转移瘤的意义尚不明确。转移性骨肉瘤患者预后是决定是否选择积极治疗模式的重要因素。原则上应平衡生存获益与治疗毒副反应或负担之间的关系^[11]^。

本研究仍存在以下不足：①本研究潜在的无应答偏倚不可忽略，调查对象同拒绝参与者之间往往存在较大差异；②问卷内容并不能涵盖真实世界中所有的诊疗模式，许多调查对象在开放性问题项目中给予了更加详细深入的回答，远远超过问题固定选项范畴；③自填式问卷调查依靠调查对象是否准确地回答问题，因此需考虑到因为有意识或无意识地顺应问题提供的答案，调查结果可能存在偏倚；④作为多学科协作实践的重要成员，参与本次问卷调查的肿瘤内科、放疗科、影像科及病理科医师数量较少，因而对于上述专业诊疗策略现况可能被低估。

总而言之，本次问卷调查提供了我国骨肉瘤肺转移诊疗模式的现况信息，反映出缺乏统一规范诊疗常规的不足。根据循证医学形成的临床指南逐年增加。对于使患者生存获益的核心诊疗策略的临床需要日益突显。以研究数据为导向可能有助于形成骨肉瘤肺转移诊疗常规，然而需要前瞻性多中心随机对照临床研究进行验证。本研究结果为今后进一步探索及制定国际临床指南提供了基线数据。

## 附录  骨肉瘤肺转移调查问卷内容

1.   您所在的医院？

A   综合性医院

B   肿瘤专科医院

C   其他_______

2.   您从事哪个专业？

A   骨科

B   胸外科

C   放疗科

D   肿瘤内科

E   病理科

F   其他_______

3.   您的工作年限？

A   0-5年

B   5-10年

C   10-20年

D   20年以上

4.   您每年诊治骨肉瘤患者数量？

A   0-10例

B   11-30例

C   31-50例

D    > 50例

5.   您对骨肉瘤肺转移的诊治原则？（单项选择）

A   个人经验

B   医院中心经验

C   文献荟萃分析

D   临床指南

E   其他_______

6.   骨与软组织肉瘤出现肺转移后，您认为哪个专业学科应主导制定治疗策略？（单项选择）

A   骨肿瘤外科

B   胸外科

C   肿瘤内科

D   放疗科

E   不确定

7.   您认为骨与软组织肉瘤肺转移的影像学评估首选哪种方法？（单项选择）

A   胸片

B   胸部CT（1 mm）

C   胸部CT（5 mm）

D   胸部MRI

E   全身PET-CT

F   不确定

8.   您认为骨肉瘤术后胸部CT随诊时间？（单项选择）

A   1个月

B   3个月

C   6个月

D   12个月

E   不确定

9.   您的经验中胸部CT诊断的准确度为多少？（单项选择）

A   30%

B   60%

C   90%

D   不确定

10.   一旦发现肺内疑似转移瘤，您是否推荐患者进行肺外转移的全身评估？（单项选择）

A   推荐

B   不推荐

C   不确定

11.   您认为骨与软组织肉瘤肺转移的最重要的治疗方案是什么？（单项选择）

A   局部治疗

B   全身治疗

C   局部- > 全身

D   全身- > 局部

E   局部+全身同时

F   其他_______

G   不确定

12.   骨与软组织肉瘤一旦出现肺内转移，您是否推荐切除肺转移瘤？（单项选择）

A   推荐

B   不推荐

C   不确定

13.   骨与软组织肉瘤一旦出现肺内转移，您是否推荐局部治疗方式？（单项选择）

A   外科手术治疗

B   影像引导消融治疗

C   立体定向放射手术（射波刀）

D   其他_______

14.   您认为肺转移瘤的最佳治疗时机是？（单项选择）

A   一旦影像学发现立即手术

B   影像学随诊3个月后手术

C   化疗XX周期后手术

D   穿刺活检确诊后手术

E   不确定

15.   您认为肺转移瘤数目超过多少个则不再适合外科治疗？（单项选择）

A   1枚

B   5枚

C   10枚

D   由胸外科医师决定

E   不确定

16.   您认为骨肉瘤肺转移术后生存获益的判断标准？（单项选择）

A   无病生存期超过1个月

B   无病生存期超过3个月

C   无病生存期超过6个月

D   其他_______

E   不确定
